# Hemodialysis Access Blood Flow and Cardiopulmonary Outcomes

**DOI:** 10.7759/cureus.98259

**Published:** 2025-12-01

**Authors:** Zahidul Mondal, Mojgan Jalalzadeh, Steve Khalil

**Affiliations:** 1 Internal Medicine/Transplant Nephrology, Rutgers Robert Wood Johnson Medical School, New Brunswick, USA; 2 Internal Medicine/Nephrology, Rutgers Robert Wood Johnson Medical School, New Brunswick, USA

**Keywords:** arteriovenous fistula, esrd life-plan, hemodialysis, high-output heart failure, left-ventricular hypertrophy, pulmonary hypertension, vascular access

## Abstract

Arteriovenous (AV) access remains the cornerstone of long-term hemodialysis, yet it profoundly alters systemic hemodynamics. The resulting increase in venous return and cardiac output is initially adaptive but, when excessive, can precipitate maladaptive cardiovascular remodeling and downstream complications. A comprehensive literature search was performed using PubMed, Scopus, and Embase databases for studies published between 2018 and 2025. The search terms included *arteriovenous fistula*, *hemodialysis*, *high-output heart failure*, *pulmonary hypertension*, and *left-ventricular hypertrophy* (LVH). Due to the heterogeneity in design and the limited availability of randomized data, this paper was developed as a narrative synthesis rather than a formal systematic review. Arteriovenous access flow exceeding 1.5-2.0 L/min, a threshold that indicates excessive flow, or representing more than 25-30% of cardiac output, is strongly associated with an increased risk of left-ventricular hypertrophy (LVH), high-output heart failure (HOHF), and pulmonary hypertension (PH). This threshold serves as a crucial clinical marker, guiding the diagnosis and management of high-flow AV access complications. Diagnostic assessment should combine duplex ultrasonography, ultrasound dilution techniques, and echocardiography. Right-heart catheterization, a procedure that involves inserting a catheter into the right side of the heart to measure pressures and oxygen levels, is used to confirm the hemodynamic burden. Optimal management involves meticulous volume control, targeted pharmacologic therapy, and, when indicated, surgical or endovascular flow reduction. Arteriovenous access remains both a therapeutic necessity and a potential hemodynamic stressor. Consistent flow surveillance, periodic echocardiographic evaluation, and individualized access planning within the ESRD Life-Plan framework, a patient-centered care model that aligns access strategy with clinical status, lifestyle, and preferences, are essential for preventing irreversible cardiopulmonary remodeling and optimizing long-term clinical outcomes.

## Introduction and background

The creation of durable vascular access, ideally an autologous arteriovenous fistula (AVF), remains fundamental to achieving hemodialysis adequacy and improving patient survival [1]. AVFs provide superior long-term patency, lower infection rates, and enhanced dialysis efficiency compared with arteriovenous grafts and central venous catheters [1,2]. Beyond their role in facilitating dialysis delivery, AVFs trigger notable systemic circulatory adaptations by diverting arterial blood directly into the venous system. This low-resistance shunt reduces systemic vascular resistance and increases venous return, leading to compensatory elevations in cardiac output mediated by both mechanical and neurohumoral mechanisms [3]. Within physiological limits, these hemodynamic adjustments are adaptive and often well tolerated. However, when access flow becomes excessive, the sustained increase in preload and cardiac output can drive chronic volume overload, eccentric left ventricular (LV) remodeling, and progressive pulmonary vascular stress. Recent observational cohorts suggest that 20-60% of hemodialysis patients develop pulmonary hypertension (PH) or related hemodynamic disturbances, reflecting the combined influence of high-flow access, diastolic dysfunction (a condition where the heart's ventricles do not relax and fill with blood as well as they should), and uremic endothelial injury [4,5]. Parallel to this, recognition of high-output heart failure (HOHF) has grown substantially, supported by newer diagnostic criteria and updated clinical frameworks, including the 2025 Cleveland Clinic algorithm for AVF-related circulatory overload. This algorithm, which provides a structured approach to identifying and managing circulatory overload in AVF patients, is a significant advancement in the field, guiding clinicians in the early recognition and management of high-flow AV access complications [6]. Growing evidence indicates that the hemodynamic effects of AV access are not merely local but systemic, influencing cardiopulmonary structure, right-heart pressures, natriuretic peptide levels, and long-term morbidity. In this context, early identification of clinically significant flow states has become increasingly important. Contemporary practice emphasizes a combination of access flow surveillance, echocardiographic monitoring, and multidisciplinary collaboration to prevent irreversible cardiac and pulmonary remodeling [7,8].

This review synthesizes current physiologic, diagnostic, and clinical evidence on how AV access flow influences cardiac and pulmonary function. It provides practical strategies for early recognition, prevention, and management of high-flow-related complications. The goal of this review is to gain a deeper understanding and know useful tools to improve patient care, providing a clear focus for the information presented.

## Review

Review approach

A thorough literature search was undertaken in the PubMed, Scopus, and Embase databases to identify relevant studies published between January 2018 and May 2025. The search terms included "arteriovenous fistula," "hemodialysis," "cardiac output," "pulmonary hypertension," and "vascular access flow." Titles and abstracts were screened for relevance to the cardiovascular and pulmonary complications associated with dialysis access, and full texts of potentially eligible studies were retrieved for detailed review. This approach ensures the validity and reliability of the information presented in the review. Original observational studies (cohort, case-control, and registry analyses), mechanistic investigations (echocardiographic, hemodynamic, and biomarker-based), and interventional reports on access-flow management were included. Non-English articles, small case-series (<10 patients) lacking quantitative cardiovascular outcomes, and studies focused solely on access patency without cardiac or pulmonary endpoints were excluded. The search strategy was supplemented by manual screening of reference lists of key review articles and by cross-checking primary recent guideline bibliographies. Because the retrieved studies exhibited substantial heterogeneity in study design, patient populations (incident vs. prevalent dialysis, graft vs. fistula vs. catheter), definitions of "high-flow" access, outcome measures (echocardiographic parameters, natriuretic peptides, right-heart catheterization data, heart failure events), and follow-up durations, a formal systematic review and meta-analysis were not feasible. Instead, a narrative synthesis was adopted, with a strong emphasis on providing clinically applicable insights and trends rather than pooled quantitative effect sizes. This approach ensures that the audience is equipped with practical knowledge that can be directly applied in their clinical practice. Evidence was grouped under key thematic domains: hemodynamic mechanisms, diagnostic assessment, cardiopulmonary outcomes, management strategies, and special populations, and the direction, consistency, and strength of findings across studies were summarized. Study quality (e.g., adjustment for confounders, measurement of access flow, and assessment of selection bias) was also critically evaluated, and knowledge gaps and areas for future research were highlighted. Limitations of the approach are acknowledged: narrative synthesis carries the risk of selective interpretation, and exclusion of non-English studies may introduce language bias. However, the incorporation of recent data spanning 2024-2025 enhances relevance to contemporary clinical practice. The clinically oriented framework supports the translation of evidence into everyday practice for nephrology, cardiology, and vascular access teams.

Hemodynamic foundations

Physiological Principles

An AVF functions as a low-resistance shunt, effectively bypassing the arteriolar capillary network and thereby reducing systemic vascular resistance while increasing venous return [9,10]. This hemodynamic alteration results in increased preload and augments cardiac output, driven by both mechanical and neurohumoral pathways [11,12]. The sudden drop in resistance and the increase in venous preload trigger the body's compensatory mechanisms, including the activation of the renin-angiotensin-aldosterone system, upregulation of sympathetic nervous system activity, and enhanced endothelin-1 release, all of which contribute to persistent volume loading and myocardial stress [10,13]. Over time, this sustained volume and pressure load stimulates LV wall stress and chamber dilation, promoting eccentric hypertrophy in accordance with the Frank-Starling mechanism (greater end-diastolic stretch leading to increased stroke volume) [14]. As the left ventricle enlarges, diastolic filling becomes impaired, left‐atrial pressure rises, and the pulmonary circulation is exposed to elevated flow and pressure. This constellation of changes predisposes to either diastolic dysfunction or, eventually, systolic failure as myocardial architecture deteriorates and contractile reserve is exhausted [15,16]. Animal and human mechanistic studies further delineate the relationship between large shunt flows and cardiac remodeling. For example, in large-animal AVF models, the creation of a high-flow shunt resulted in an increase in cardiac output, sustained reductions in systemic vascular resistance, and early signs of LV volume loading [17]. In patients on hemodialysis, high-flow accesses (often exceeding 1.5-2.0 L/min) have been repeatedly associated with an increase in LV mass index, reduced ejection fraction, and worsening natriuretic peptide profiles [14]. Clinically, these physiological changes translate into a spectrum of cardiovascular sequelae: persistent excessive AVF flow may accelerate left ventricular hypertrophy (LVH), promote atrial enlargement, precipitate arrhythmias, and contribute to the development of HOHF or PH. Identifying patients in the early phase of this maladaptive remodeling offers a therapeutic window to intervene, either by addressing flow burden, optimizing volume status, or modulating neurohumoral influences, before irreversible cardiac damage occurs. This potential for intervention underscores the importance of early detection and management in clinical practice.

In summary, while AVFs remain the preferred long-term hemodialysis access due to improved patency and reduced infection risk, their systemic hemodynamic impact warrants vigilant evaluation. The interplay of increased venous return, reduced vascular resistance, and chronic volume loading forms the mechanistic basis by which high-flow AVFs impose cardiovascular stress. Recognizing this subtle pathophysiologic burden is crucial for linking access planning and surveillance to long-term cardiovascular outcomes in patients with end-stage renal disease (ESRD). These considerations reinforce the role of integrated access-cardiovascular surveillance in patients with ESRD.

Defining High Flow 

The concept of "high flow" in the context of arteriovenous access remains variably defined in the literature. Many investigators and clinical guidelines cite a threshold access flow (Qa) of ≥1.5 to 2.0 L/min or a Qa-to-cardiac output ratio (Qa/CO) of ≥0.25 to 0.30 as representing elevated hemodynamic burden. Studies describe that once these thresholds are crossed, patients are more likely to experience LV dilation, elevated pulmonary artery pressures, and symptoms associated with HOHF [10]. Nevertheless, a lack of universally accepted criteria persists. For example, the Vascular Access Society defines a "high-flow" fistula as one with a Qa of 1.0-1.5 L/min or approximately 20% of cardiac output [18]. Other investigations have adopted more conservative cut-offs or factored in access size, patient cardiac reserve, and the rate of flow increase over time [9]. Due to this heterogeneity, establishing standardized diagnostic benchmarks for high-flow access is not only important but also crucial, as it enables reproducible research, facilitates meaningful comparisons across studies, and promotes standardized clinical decision-making. From a mechanistic point of view, the threshold is critical because once the AV access shunt approaches or exceeds this high-flow range, the circulatory system is challenged. The shunt diverts a significant proportion of cardiac output into a low-resistance circuit, thereby reducing effective systemic vascular resistance and further increasing cardiac preload. This scenario causes ventricular volume loading and increased right-heart and pulmonary vascular stress, setting the stage for structural remodeling and cardiopulmonary complications. In practical terms, measurement of access flow alone is insufficient. Best practice involves correlating Qa with cardiac output (ideally via echocardiography or other hemodynamic assessment) to compute the Qa/CO ratio, thereby gaining a more meaningful evaluation of the shunt burden relative to the patient's overall cardiac capacity. In this way, access flow metrics may be interpreted in the context of each patient's cardiac reserve, comorbidities, and duration of dialysis exposure.

In summary, although exact numeric thresholds continue to differ, the consensus emerging from observational and mechanistic studies is that access flows above approximately 1.5-2 L/min or representing more than approximately 25-30% of cardiac output carry a significantly increased risk of ventricular remodeling and cardiopulmonary sequelae. Until harmonized definitions are adopted, clinicians should interpret access flow within the broader clinical context. In the absence of standardized criteria, additional indicators such as echocardiographic changes, symptoms of volume overload, and biomarkers of cardiac stress play a vital role in clinical evaluation. These indicators, combined with clinical judgement, contribute to a comprehensive assessment of patient status.

Effect of Access Configuration

The anatomical site of vascular access significantly influences the magnitude of flow and, therefore, the hemodynamic burden experienced by the patient. In published observational series, distal radiocephalic AVFs, typically wrist-based, generate flow rates in the range of approximately 600-1,200 mL/min. In contrast, proximal brachiocephalic or basilic AVFs (upper arm) often exceed 1,500 mL/min. For example, a 2025 study found mean flows of approximately 0.85 L/min for distal fistulas versus approximately 1.47 L/min for proximal fistulas, with significantly higher cardiac output and cardiopulmonary recirculation observed in the proximal group [19]. Although proximal fistulas tend to mature more reliably and provide higher dialysis adequacy, they carry an increased hemodynamic burden: elevated venous return, larger shunt volumes, and greater cardiac preload. These factors are associated with a higher incidence of LV dilatation, elevated right-heart pressures, and an increased risk of PH. In one mechanistic report of a large proximal AVF, temporary occlusion resulted in a marked reduction in cardiac output and pulmonary artery pressure, highlighting the direct impact of flow from proximal access on cardiopulmonary dynamics [10]. From a clinical perspective, the choice of access configuration should account not only for ease of cannulation and patency but also for long-term cardiovascular risk. It is crucial to consider a comprehensive decision-making framework that integrates access-site selection into the broader context of patient care, such as an ESRD Life Plan, which balances access durability with cardiovascular stability. This approach is supported by cumulative evidence and can help optimize patient outcomes.

Clinical sequelae: cardiac and pulmonary manifestations

LV Remodeling

Excessive arteriovenous access flow places a sustained preload burden on the left ventricle. The persistent increase in venous return triggers adaptive myocardial hypertrophy, initially eccentric in nature, as the left ventricle dilates to maintain stroke volume and ejection fraction under conditions of volume overload. Over time, this adaptive hypertrophy becomes maladaptive: LV wall stress and chamber expansion lead to impaired relaxation (diastolic dysfunction), followed by dilatation and eventual systolic dysfunction in susceptible patients [14,20,21]. In hemodialysis populations, imaging studies have reported associations between elevated access flows and increasing LV mass index, left atrial volume, and NT-proBNP levels. For example, one study using cardiac magnetic resonance imaging (MRI) demonstrated a mean increase of approximately 7 g in LV mass after AVF creation in patients with flows >600 mL/min, compared with minimal change in the lower-flow group [20]. A recent synthesis reported that patients with high-flow fistulas had more frequent LV dilatation and remodeling than those with standard flows [22]. These structural heart changes carry clinically meaningful consequences. LV hypertrophy (LVH) is a well-recognized independent predictor of cardiovascular events and mortality in chronic kidney disease and dialysis cohorts. The combination of LVH, left-atrial enlargement, increased filling pressures, and neurohormonal activation underpins the development of arrhythmias (especially atrial fibrillation), heart failure with preserved ejection fraction, and eventual HOHF [23,24]. To prevent these adverse outcomes, it is crucial to integrate monitoring tools such as serial echocardiography (assessing LV mass index, left atrial volume index, and E/e′ ratio [E = early diastolic mitral inflow velocity; e′ = early diastolic mitral annular velocity]) and biomarkers such as NT-proBNP into access-flow surveillance. Addressing elevated access flows early, before irreversible myocardial fibrosis or maladaptive remodeling takes hold, may reverse or attenuate these changes. Indeed, studies of AVF ligation or flow reduction have demonstrated reductions in LV mass and improved functional status [20,25,26].

HOHF in the Dialysis Setting

HOHF is defined as a clinical syndrome of heart failure symptoms (e.g., dyspnea, fatigue, orthopnea, peripheral edema) occurring in the context of elevated cardiac output. The development of HOHF in patients with an AVF is a significant concern, as the shunt flow imposes a chronic hyperdynamic circulatory burden. This low-resistance access leads to increased venous return and reduced systemic vascular resistance, resulting in persistently elevated cardiac output, a state that gradually exhausts myocardial reserve. Clinically, hemodynamic assessment (e.g., right-heart catheterization or transient manual AVF compression during echocardiography) may support the role of the access: occlusion of the AVF often results in an immediate drop in cardiac output and filling pressures [18]. Observational cohort studies of dialysis access populations report baseline cardiac outputs in the range of 7-8 L/min (with shunt flows >2 L/min) and subsequent symptomatic improvement following access-flow reduction [22]. In this setting, patients may present with heart failure symptoms, even in the presence of preserved ejection fraction, unless the contribution of high-flow vascular access is recognized. Manifestations may include elevated NT-proBNP levels, progressive LV dilatation, elevated pulmonary pressures, right-heart strain, and declining exercise tolerance. Early recognition is important because the underlying driver (the shunt) is modifiable. Addressing the AVF's flow burden, optimizing volume status, and mitigating neurohumoral activation may help prevent irreversible cardiac damage. From a management standpoint, identifying patients with elevated access flows and signs or symptoms of HOHF should prompt a collaborative, multidisciplinary evaluation of the AVF. This team-oriented approach is crucial for developing and implementing potential strategies, which may include flow reduction interventions (such as banding, plication, or ligation), optimizing dry weight and volume status, and conducting a detailed cardiac assessment. Because the access itself can drive the hyperdynamic state, addressing it may lead to significant hemodynamic improvement and symptomatic relief.

In summary, HOHF represents a potentially reversible form of heart failure in dialysis patients with high-flow arteriovenous access. Recognizing its characteristic hemodynamic signature, elevated cardiac output and associated symptoms, and integrating access-flow surveillance into the care pathway may enable earlier identification of at-risk patients and timely intervention to prevent long-term cardiopulmonary sequelae. The possibility of reversibility underlines the importance of vigilant monitoring and proactive management in this patient population.

​*Pulmonary Hypertension*

PH in hemodialysis patients arises via multiple overlapping mechanisms. It is crucial to understand that early detection, achieved through echocardiographic surveillance and biomarker monitoring, is key to managing this condition. This proactive approach can significantly improve long-term outcomes. First, increased pulmonary blood flow, often secondary to a high-flow AVF or elevated cardiac output state, places a volume load on the pulmonary circulation and contributes to vascular remodeling. Second, post-capillary congestion from LV diastolic dysfunction and elevated left-atrial pressure further elevates pulmonary venous and capillary pressures, promoting pulmonary vascular remodeling and right-heart strain. Third, a milieu of uremic endothelial injury, systemic inflammation, vascular calcification, and micro-embolic phenomena further injures the pulmonary vascular bed [19]. In patients undergoing hemodialysis, the prevalence of PH is substantial: Some echocardiographic studies report that 30-50% of patients have elevated pulmonary artery pressures. Significantly, the hemodynamic perturbations introduced by a high-flow AVF, increased flow, reduced systemic vascular resistance, and augmented venous return, further amplify this risk and make the dialysis access context a unique facilitator of pulmonary vascular stress [16]. From a pathophysiologic perspective, the progression typically begins with elevated right-ventricular preload and mildly increased pulmonary artery pressures. Over time, the pulmonary circulation adapts: Initial vasodilation gives way to smooth muscle hypertrophy, vascular stiffening, and reduced vascular reserve. Chronic hyperdynamic flow, repetitive volume shifts during dialysis sessions, and micro-embolism exposure accelerate remodeling. The increased afterload on the right ventricle then fosters hypertrophy, wall stress, and eventual right-heart failure. Clinically, this has significant implications. PH in the hemodialysis population is associated with increased mortality, reduced exercise capacity, and greater cardiovascular complications. Early detection may be achieved via echocardiographic surveillance (e.g., measuring tricuspid regurgitation velocity, right atrial and right ventricular dimensions, and pulmonary artery systolic pressure) and biomarker monitoring (e.g., NT-proBNP and troponin). When indicated, invasive hemodynamic assessment may enable identification of at-risk patients before overt right-heart failure develops. In the context of a high-flow AVF, recognition of early signs of pulmonary vascular stress, rather than waiting for symptomatic right-heart failure, provides a timely opportunity for intervention. Strategies may include AVF flow reduction, optimization of volume status, and aggressive management of left-heart diastolic dysfunction and volume overload.

In summary, PH in the hemodialysis cohort is a multifactorial complication in which the hemodynamic burden of high-flow vascular access plays an under-recognized role. By incorporating these insights into vascular access planning and longitudinal surveillance, the audience, including nephrologists, cardiologists, dialysis clinicians, and medical researchers, plays a crucial role in mitigating the progression of cardiopulmonary sequelae and improving long-term outcomes. This emphasis on their integral role will make them feel empowered and responsible for the management of PH.

Clinical implications of LV remodeling in hemodialysis access

LVH and chamber dilatation resulting from high-flow AVFs are clinically significant findings. Cardiac remodeling in this context carries several important implications.

Progression to Heart Failure and Arrhythmia

Sustained volume overload from an AVF promotes myocardial fibrosis and diastolic dysfunction, which may precede overt systolic failure. Recent data demonstrate that higher access flows are associated with an increased incidence of heart failure symptoms, reduced ejection fraction, and elevated cardiac biomarkers [9].

Elevated Cardiovascular Mortality Risk

In patients with chronic kidney disease (CKD) and ESRD, LVH is a well-established independent predictor of cardiovascular events and mortality. The additional remodeling triggered by high-flow access may accelerate this underlying risk and hasten cardiovascular decline [25].

Interaction With Pulmonary Vascular Burden

LV remodeling elevates left-atrial pressures and contributes to pulmonary venous congestion, thereby increasing the risk of PH. In dialysis patients with high-flow AVFs, studies have shown associations with elevated pulmonary artery pressures and right-heart strain [14].

Reversible Remodeling

A beacon of hope. It is crucial to understand that remodeling in this context is not a one-way street. Interventions such as AVF ligation or flow reduction in transplant recipients or dialysis patients have been shown to reverse LV mass, improve NT-proBNP levels, and alleviate symptoms [11]. This reversibility underscores the importance of early detection and proactive management.

Comprehensive Care

The role of access planning and surveillance. Given the potential for cardiovascular remodeling, access flow should not be viewed solely through the lens of dialysis adequacy. The long-term cardiovascular impact also merits consideration. Therefore, routine access-flow monitoring, echocardiography for LV mass and atrial volumes, and biomarker tracking should be integrated into vascular access programs [11,16]. This comprehensive approach ensures that all aspects of patient care are considered and managed effectively.

Risk Stratification

High-flow AVF can act as a second hit. Patients with pre-existing LVH, coronary disease, or diminished cardiac reserve are particularly vulnerable to the added hemodynamic burden of a high-flow AVF. In such individuals, distal access sites, lower-flow configurations, or pre-emptive flow moderation may be appropriate to mitigate risk [10,22]. 

In summary, early recognition is a key to effective management. The development of LV remodeling in patients with high-flow arteriovenous access serves as both a marker and a mediator of cardiovascular risk. It illustrates the intersection of vascular access engineering and cardiovascular physiology. Recognizing the remodeling process early offers an opportunity for vigilance and early intervention, which are crucial in managing the cardiovascular implications of high-flow AVFs.

Diagnostic evaluation: access-flow Measurement

Quantifying vascular access flow (Qa) and assessing the hemodynamic burden of arteriovenous (AV) accesses in hemodialysis involve several techniques. Each method has its own advantages and limitations, and they all play a crucial role in the multidisciplinary management of patients on dialysis. Healthcare providers are key players in this collaborative evaluation process, and their involvement is essential for successful patient care.

Ultrasound Dilution Technology

Widely used during dialysis sessions, ultrasound dilution methods (e.g., the Transonic system) enable rapid intradialytic Qa measurements, providing quick and efficient results. In a recent observational study of 65 hemodialysis patients, the Transonic system reported a mean Qa of 1,413 ± 715 mL/min and demonstrated reproducibility, with 80% of paired measurements taken on different days remaining within ±25% of each other [20]. This modality offers the practical advantage of serial monitoring during routine sessions and provides a reliable tool for trend tracking.

Duplex Doppler Ultrasonography

Duplex ultrasonography remains a fundamental technique for simultaneous anatomical and hemodynamic evaluation of vascular access. It allows visualization of vessel diameter, flow velocity, high-velocity jets, stenosis, dilatation, and collateral circulation. Although operator-dependent and more time-consuming than dilution methods, it contributes valuable structural detail that complements pure flow measurement.

Phase-Contrast Magnetic Resonance Imaging

Phase-contrast MRI provides high-precision quantification of both access flow and cardiac output. For example, studies using flow-sensitive MRI sequences in AVFs have reported hemodynamic differences according to anatomic configuration and maturation status [21]. Nevertheless, the utility of MRI is limited to specialized centers due to cost, logistical constraints, and the need for technical expertise, rendering it impractical for routine surveillance in most dialysis units.

Right-Heart Catheterization

Although invasive, RHC remains a comprehensive and gold standard method for cardiopulmonary hemodynamic assessment, particularly when elevated pulmonary vascular resistance, right-heart dysfunction, or a suspected high-output state is present. In dialysis access populations, RHC studies have identified pre-capillary PH in approximately one-third of patients even after volume optimization [22]. While not suited for routine monitoring, RHC provides critical diagnostic clarity in selected cases, especially when flow reduction interventions are being considered, giving healthcare providers a comprehensive tool for diagnosis.

Integration of Measurements and Clinical Context

It is crucial in the interpretation of access-flow data. Access-flow data should be interpreted in the context of each patient's overall clinical status. The implications of Qa depend on broader contextual factors, including patient symptoms, echocardiographic findings, biomarker levels, and a history of volume status and dialysis adequacy. For instance, a Qa of 1.8 L/min may be well tolerated in a younger patient with robust cardiac function, yet may trigger remodeling in an older patient with impaired ventricular reserve. In practice, integrating flow measurement into the overall cardiovascular assessment is essential for longitudinal surveillance and timely intervention, underscoring the significance of healthcare providers' role in patient care.

Surveillance of vascular access flow

Routine surveillance of vascular access flow (Qa) and related cardiovascular parameters is increasingly recognized as a key element in the long-term management of patients on hemodialysis, particularly those with high-flow arteriovenous (AV) accesses. The National Kidney Foundation's KDOQI 2019 Vascular Access Guidelines state that "regular physical examination of the access and surrounding limb before every cannulation" is advisable (Statement 13.1) and that such checks are "reasonable" for all dialysis facilities to detect access-flow dysfunction (Statements 13.1-13.3) [1]. Although the Guidelines stop short of endorsing routine quantitative Qa measurement for every patient, citing "inadequate evidence" (Statements 13.4-13.5), emerging observational studies and expert opinions suggest that access-flow monitoring may extend beyond patency alone to include cardiovascular risk assessment.

In operational terms, a comprehensive surveillance programme begins with a baseline assessment after access maturation (typically 4-6 weeks post-creation), followed by periodic monitoring of Qa (e.g., every three to six months, or sooner if new cardiopulmonary symptoms such as dyspnea, orthopnea, rising central venous pressure, or unexplained decline in cardiac function appear). Additional criteria for cardiovascular evaluation may include Qa exceeding approximately 2 L/min or a Qa/CO (access flow : cardiac output) ratio surpassing approximately 0.25 (some studies suggest risk may increase when Qa/CO > 0.30). Longitudinal trend analysis is of particular importance: A rapid increase in Qa, a rising Qa/CO ratio, or new echocardiographic or biomarker abnormalities may warrant further investigation, even if absolute values remain below classical thresholds.

Standardized measurement conditions enhance reliability and reduce variability; assessments should be performed under consistent conditions such as pre-dialysis timing, stable ultrafiltration volume, consistent blood-pump flow, and normalized volume status (i.e., stable fluid balance). Flow surveillance must also be interpreted in the context of cardiopulmonary assessment: echocardiographic findings (such as LV mass index, atrial volumes, estimated pulmonary pressures), biomarkers (e.g., NT-proBNP), and clinical symptoms must all be considered. When elevated flows or evolving cardiopulmonary changes are detected, it is appropriate for a multidisciplinary team, including nephrology, cardiology, and vascular access intervention specialists, to evaluate the case. Their collective expertise is vital in deciding on possible actions, which may include intensified monitoring, medical optimization (volume, blood pressure, anemia), and access-flow reduction interventions (such as banding or distal revision). The audience's role in maintaining structured surveillance logs that link access flow, cardiac metrics, and clinical outcomes (e.g., heart failure events or hospitalizations) is crucial, as it enables continuous quality improvement and adaptation of thresholds to their patient population.

In summary, vascular access flow surveillance in hemodialysis patients is evolving from a narrow focus on patency to a broader cardiovascular and hemodynamic paradigm. High-flow accesses may represent an often-reversible hemodynamic stressor. The early identification of these stressors through structured surveillance offers a practical pathway to mitigate long-term cardiopulmonary complications, empowering healthcare professionals to take a proactive role in patient care.

Special populations

Elderly and Frail

In older or medically frail individuals, cardiovascular reserve is frequently diminished. The hemodynamic burden imposed by a high-flow AVF, characterized by increased cardiac output and reduced systemic vascular resistance, may therefore carry a disproportionate risk. In such patients, this burden may amplify the likelihood of heart failure, myocardial ischemia, and arrhythmias [23,24].

The concept of a patient-centered "ESRD Life-Plan" is not just a framework, but a guiding light in tailoring vascular access decisions to a patient's prognosis, functional status, and comorbidity profile [1]. For patients with an anticipated survival exceeding two years and preserved functional capacity, a distal AVF (e.g., a wrist radiocephalic AVF) may represent a preferred access configuration. Created in the forearm, this configuration typically imposes less hemodynamic burden and is associated with fewer cardiovascular complications compared with more proximal AVFs, making it a favorable long-term option [25].

For patients with limited life expectancy, significant frailty, or multiple comorbidities, alternative approaches, including an arteriovenous graft (AVG) or tunneled catheter, may better align with priorities of minimizing procedural risk, hospitalization, and flow-induced cardiovascular stress [26]. The use of preoperative frailty-screening tools (such as PRISMA-7, the Fried phenotype, or a frailty index) is not just a suggestion, but a crucial step that aids clinicians in identifying patients less likely to benefit from high-flow fistula creation, thereby guiding more nuanced access planning [27].

In summary, vascular access strategy in the elderly or frail involves balancing dialysis adequacy, access durability, and long-term cardiovascular stability. However, it's crucial to remember that framing access planning within a holistic, patient-centered "ESRD Life-Plan" is not just a step, but a cornerstone that helps ensure decisions reflect the individual’s overall health trajectory and goals.

Post-transplant

After successful kidney transplantation, a persistent AVF may continue to function as a systemic shunt, leading to a hyperdynamic circulatory state. This can put a strain on the patient's cardiovascular system, even when renal function has been restored. Several studies have shown that elective ligation or flow reduction of a patent AVF in stable transplant recipients is associated with improvements in cardiac structure and function, including reductions in LV mass, cardiac output, and atrial volumes [25,28]. For example, a randomized trial reported a mean decrease in LV mass of 22.1 g at six months after AVF ligation compared with minimal change in the control group, alongside reductions in cardiac output, NT-proBNP, and atrial volumes [25]. Observational work further suggests that flow reduction interventions (rather than complete ligation) in post-transplant patients with high-flow AVFs are linked to symptomatic improvement, a lower cardiac index, and improved renal parameters [29].

Clinical decision-making in this context involves balancing the preservation of vascular access for possible dialysis return against long-term cardiovascular risk. Essential factors include allograft function, projected graft longevity, AVF flow magnitude (for instance, flows exceeding approximately 1.5-2.0 L/min), evidence of cardiac remodeling or symptoms consistent with a high-output state, and the patient's overall comorbidity burden. In practice, a multidisciplinary team, including transplant nephrology, vascular access specialists, and cardiology, may convene to evaluate the benefits and risks of AVF ligation or flow-reduction in the post-transplant setting.

When graft function stabilizes, a structured evaluation is advised. The first step is to confirm stable allograft status (e.g., more than 12 months post-transplant, stable creatinine levels, and no recent rejection). Next, vascular access flow (Qa) should be measured and a comprehensive cardiac evaluation performed (including echocardiography assessing LV mass, atrial volumes, and optionally NT-proBNP). Simultaneously, the patient should be evaluated for signs or symptoms of hemodynamic burden such as dyspnea on exertion, orthopnea, elevated cardiac output indices, or raised pulmonary artery pressure. Once this information is obtained, risk stratification follows. Patients with Qa below approximately 1.2-1.5 L/min, without symptoms and with standard cardiac structure, can continue routine surveillance. Those in the intermediate-risk category (Qa around 1.5-2.0 L/min, early cardiac changes, and minimal symptoms) warrant closer monitoring and cardiology review with consideration of flow reduction options. Patients at higher risk (Qa ≥2.0 L/min or Qa/CO >0.25, evidence of cardiac remodeling, symptoms of a high-output state, or PH) may require active intervention planning.

If dialysis return seems unlikely, elective access ligation may be appropriate. If the access may still be needed but the flow is excessive, flow reduction strategies (such as banding, plication, or distal revision) might be preferred over complete ligation. After intervention, a follow-up cardiac assessment at approximately three to six months, including repeat echocardiography, NT-proBNP, remeasurement of Qa, and symptom reassessment, is recommended. Access surveillance should continue at three- to six-month intervals to ensure that the AVF remains within the desired flow range and to detect any potential issues early. Cardiopulmonary monitoring annually or sooner if symptoms arise is recommended to assess the patient's overall cardiovascular health. Outcome monitoring remains critical: changes in LV mass, cardiac output, symptom burden, and hospitalization for cardiovascular events should guide adjustment of access management and cardiac surveillance. If evidence of cardiac remodeling reversal and clinical stability is achieved, transition to routine care may be appropriate; if not, further intervention should be reconsidered.

Younger and Pediatric

Younger patients receiving hemodialysis, especially those with large-caliber proximal arterial anastomoses for permanent vascular access, are at increased risk of excessive arteriovenous (AV) flow and associated hyperdynamic circulation. Because of their smaller body size and heightened cardiac sensitivity, even modest absolute access flows may lead to a disproportionately high Qa/CO (access flow to cardiac output) ratio, thereby accelerating ventricular remodeling (the process of changes in the size, shape, and function of the heart) [10].

Early and periodic measurement of access flow (Qa) is critical in this group. Monitoring allows detection of evolving high-output states or pre-clinical indicators of cardiomyopathy, offering an opportunity to prevent ventricular dilation or dysfunction. What's more, the regular use of non-invasive cardiac imaging (e.g., echocardiography) and biomarkers of cardiac stress plays a crucial role in providing a comprehensive diagnostic approach for managing these patients.

In pediatric access creation, strategies, such as favoring more distal, lower-flow configurations (e.g., basilic vein transposition or brachiocephalic fistula) where vascular anatomy permits, and incorporating longitudinal surveillance into care planning, may help mitigate the risk of flow-mediated cardiac sequelae.

In summary, younger or pediatric hemodialysis patients warrant a surveillance paradigm that is sensitive to access-flow dynamics. It is crucial to recognize the unique hemodynamic vulnerability of developing cardiovascular systems. This understanding supports a structured and proactive management approach in this population.

Management strategies

Non-procedural Optimization: Intradialytic Volume Management

Meticulous management of intradialytic volume removal is foundational to reducing cardiovascular stress in hemodialysis patients. Achieving and maintaining an accurate dry weight, which is the patient's weight when they have no excess fluid in their body, while keeping ultrafiltration rates (UFR) below approximately 13 mL/kg/h, constitutes a key component of patient care. This approach has been associated with fewer disturbances in myocardial perfusion and reduced LV strain [30]. Healthcare professionals play an integral role in achieving and maintaining this accurate dry weight, which supports patient well-being in hemodialysis settings.

Simultaneously, optimal treatment of comorbid conditions, including anemia, hypertension, and obstructive sleep apnea, significantly contributes to lowering cardiovascular load and improving hemodynamic stability. Given the cumulative cardiovascular burden posed by repeated fluid removal, it is essential to optimize the hemodynamic milieu through non-procedural means. Such an approach supports alignment of procedural vascular access interventions with a foundation of volume and cardiovascular optimization, which may contribute to improved care decisions.

Pharmacologic Adjuncts

Adjunctive pharmacological strategies contribute to both vascular access function and cardiovascular stability in patients undergoing hemodialysis. For example, antiplatelet therapy (such as aspirin alone or combined with dipyridamole) has been shown to reduce graft thrombosis risk and prolong primary unassisted patency in new arteriovenous grafts (hazard ratio 0.82; 95 % confidence interval 0.68-0.98; P = 0.03) [31]. Omega-3 fatty acid supplementation (fish oil or ω-3 polyunsaturated fatty acids) has demonstrated anti-inflammatory and endothelial-stabilizing effects and has been linked in some studies to lower thrombosis and intervention rates in vascular access; however, the overall benefit to patency remains uncertain [32]. In addition, agents such as angiotensin-converting enzyme inhibitors (ACE-Is), angiotensin receptor blockers (ARBs), and calcium channel blockers (CCBs) may enhance endothelial function, reduce systemic afterload, and are associated in observational data with modest improvements in access longevity and cardiovascular stability.

Surgical and Endovascular Flow Reduction

When a high-flow AVF leads to persistent HOHF or PH despite optimized medical management, surgical or endovascular flow-reduction interventions should be considered. Techniques such as banding, plication, angioplasty of inflow or outflow segments, and revision procedures including distal-inflow redesign (for example, the Revision Using Distal Inflow [RUDI] procedure, which involves creating a new inflow site to reduce flow) aim to reduce shunt flow while preserving dialysis adequacy [6]. Complete ligation of the access is generally reserved for refractory cases or transplant recipients in whom the AVF is no longer needed [33]. Controlled reduction of AVF flow (Qa) has been shown to lower cardiac output, reduce pulmonary artery pressures, and reverse or halt remodeling of both left and right ventricles while maintaining effective dialysis access [34]. Clinical decision-making should integrate the patient’s cardiovascular status, residual cardiac reserve, access anatomy, dialysis requirement, and target flow thresholds. This evaluation ideally occurs within a multidisciplinary forum, including nephrology, vascular access surgery/intervention, and cardiology teams, to ensure coordinated decision-making.

Preventive planning

Preoperative duplex (or color) Doppler ultrasound mapping provides a foundational preventive strategy in the creation of arteriovenous (AV) access. This imaging modality evaluates both arterial and venous anatomy, including lumen diameters, wall integrity, accessory veins, and vessel distances, to optimize access site selection and minimize subsequent hemodynamic burden. A preference for more distal access sites (e.g., wrist or forearm fistulas) rather than proximal upper-arm configurations may reduce shunt volume, preserve proximal vascular real estate, and thereby lessen the risk of high-flow states and downstream cardiac or pulmonary stress [1]. Intraoperative control of anastomotic diameter and configuration, by avoiding overly broad arterial anastomoses, ensuring a balance of venous inflow and outflow, and limiting unnecessary collateral branches, further contributes to reducing the risk of excessive flow and a hyperdynamic circulation [26]. Together, vessel mapping, distal-site preference, and controlled anastomosis form a preventive framework that addresses the hemodynamic consequences of AV access from the outset, enabling more proactive clinical management.

Monitoring and follow-up

Comprehensive and routine monitoring of patients with AV access is a key component of an integrated multidisciplinary care model. This model, which includes access surveillance, routine measurements of access flow (Qa), and a detailed physical examination of the access site and limb, is designed to evaluate stenosis, aneurysmal changes, abnormal inflow or outflow, and collateral formation. In higher-risk patients, such as those with elevated access flows, evidence of cardiac remodeling, or suspected PH, cardiopulmonary monitoring becomes critical. In these cases, annual echocardiography should assess LV and right-ventricular geometry, chamber volumes, and estimated pulmonary pressures; further imaging or right-heart catheterization may be indicated when symptoms or abnormal flow patterns emerge. This close coordination among nephrology, cardiology, and vascular access teams is a testament to our commitment to providing comprehensive care, ensuring the timely identification of evolving cardiopulmonary stress, informed decision-making between access function and cardiovascular stability, and appropriate intervention planning.

Future directions

As we look to the future, there are several key areas where further research is needed to advance our understanding and management of AV access. Standardization of flow definitions is a crucial step, as the current literature employs variable thresholds for access flow (Qa) and the ratio of access flow to cardiac output (Qa/CO), which limits comparability across studies and clinical practice. Larger prospective outcome trials are also required, as there is a marked paucity of randomized, longitudinal studies assessing survival, cardiovascular hospitalizations, and long-term outcomes of flow reduction interventions in high-flow AV accesses. Advanced risk-prediction tools, including machine learning algorithms, hold promise in identifying patients at the highest risk of developing high-flow states, cardiovascular remodeling, or PH, enabling proactive intervention rather than reactive management. A mechanistic investigation into uremic endothelial dysfunction, pulmonary vascular remodeling, and neurohumoral responses to high-flow arteriovenous shunts is needed to refine patient stratification and enable the development of novel therapeutics [35,36]. Finally, given the distinct physiological reserve, comorbidity burden, and functional goals of older adults on hemodialysis, targeted research comparing distal versus proximal access configurations, flow thresholds adapted to less resilient cardiovascular systems, and quality-of-life/functional outcome frameworks is required [37]. These future research directions offer hope for significant advancements in AV access management.

Discussion

The hemodynamic consequences of arteriovenous access in dialysis extend far beyond concerns about needle access or pump flow. When a large-flow AVF is created, the cardiovascular system experiences a persistent volume-overload state: venous return increases, cardiac output rises, and the left ventricle adapts through dilation and hypertrophy. Over time, this compensatory remodeling may transition into a maladaptive phase, leading to dysfunction, heart failure, and heightened cardiovascular risk [16]. A window for early recognition and intervention exists. Well before overt heart failure becomes clinically manifest, structural changes, such as increased LV mass, left-atrial enlargement, and pulmonary vascular remodeling, may be detectable. Thus, routine surveillance of access flow (Qa), combined with periodic echocardiographic evaluation and biomarker assessment, may support a strategy for identifying patients at risk and intervening before irreversible myocardial fibrosis or right-heart failure arises [14].

In patient populations characterized by advanced age, frailty, or reduced cardiac reserve, the hemodynamic burden of a high-flow AVF may tip the balance unfavorably. An access configuration suitable for a younger patient with preserved cardiovascular reserve may impose excessive burden on a frail individual. In such settings, prioritizing cardiovascular stability and, where appropriate, selecting a lower-flow access configuration aligns with a patient-centered care framework. This highlights the importance of collaboration across specialties: optimal decision-making requires input from nephrology, cardiology, and vascular surgery to develop an individualized plan that harmonizes dialysis access goals with cardiovascular risk, functional status, and life expectancy. The concept of an "ESRD Life-Plan," a comprehensive care plan that takes into account the patient's broader health trajectory, including their dialysis access and cardiovascular health, supports coordinated care and patient-centered decision-making.

Nevertheless, challenges remain. There is no universally accepted definition of "high flow" access; most data derive from observational cohorts, and randomized trials of access-flow reduction or modulation remain sparse. Clinicians, therefore, often operate in a context of informed uncertainty, tracking trends, integrating imaging and hemodynamic data, and reviewing access strategy when the hemodynamic burden becomes evident. In practice, surveillance and dynamic reassessment are essential. Through monitoring access flow, correlating this with cardiac imaging and biomarkers, and remaining alert for subtle signs of cardiac remodeling or pulmonary vascular stress, providers may intervene earlier. Pairing this surveillance with access planning tailored to each patient's cardiovascular profile offers a pathway toward achieving both dialysis adequacy and long-term cardiovascular health.

Limitations

This review is constrained by several key challenges in the existing literature. First, the definitions of "high-flow" vascular access vary significantly: studies use different thresholds for access flow (Qa) or Qa/CO ratios, which hinders direct comparability across investigations. Second, the evidence base is dominated by observational studies rather than randomized controlled trials (RCTs), limiting the strength of causal inferences regarding timing, effectiveness, and long-term impact of flow-reduction interventions. Third, the absence of large-scale RCTs focusing on access-flow modification and cardiovascular outcome endpoints in dialysis populations remains a significant evidence gap. Although the inclusion of recent publications from 2024 to 2025 enhances contemporary relevance, these limitations must temper the strength of conclusions. It is important to note that while the review provides valuable insights, the limitations in the current literature highlight the need for further research and the potential for evolving understanding in this area.

Clinical Highlights 

High-flow AVFs, commonly defined as access flows exceeding approximately 2 L/min, are increasingly recognized as reversible causes of HOHF and PH; routine measurement of access flow (Qa), when integrated with periodic echocardiographic and pulmonary hemodynamic surveillance, may enable earlier detection of evolving cardiac or pulmonary remodeling, potentially before symptom onset; when clinically indicated, flow-reduction procedures (e.g., banding, plication, or surgical revision) may restore near-normal hemodynamics while preserving dialysis adequacy, thereby mitigating long-term cardiovascular risk; the "ESRD Life-Plan" framework emphasizes individualized vascular access planning and interdisciplinary coordination among renal, cardiology, and vascular specialists to support cardiovascular stability and optimize long-term outcomes; emerging research priorities include standardization of "high-flow" definitions, development of predictive analytics to identify high-risk patients, and execution of prospective trials of access-flow modification to translate pathophysiologic insights into clinical practice.

## Conclusions

The creation and maintenance of vascular access for hemodialysis is both indispensable and physiologically demanding. AVFs, although the preferred access type for their durability and low infection risk, exert measurable systemic effects. Elevated access flow, especially in proximal configurations, can produce persistent volume overload, predisposing patients to LVH, high-output heart failure, and pulmonary hypertension. Evidence consistently shows that high-flow AVFs correlate with worsening cardiac structure and function. Importantly, these complications are not predetermined; when excessive flow states are identified early, the resulting cardiopulmonary changes can often be halted or partially reversed. This underscores the crucial role of routine flow surveillance (including Qa and Qa/CO ratios) in preventing irreversible cardiopulmonary changes, along with regular echocardiography, cardiopulmonary biomarker monitoring, particularly among high-risk individuals.

Integrating multidisciplinary expertise is crucial for balancing dialysis adequacy with cardiovascular safety. Coordinated decision-making among nephrology, vascular access specialists, and cardiology is of paramount importance, as it establishes a framework for early intervention and the prevention of irreversible remodeling. Incorporating the "ESRD Life-Plan" helps align access strategy with each patient's broader clinical trajectory, functional status, and life goals. For older or frail patients, preserving cardiac reserve and quality of life may take precedence over maximizing access longevity, prompting individualized choices regarding access configuration, flow targets, and monitoring intensity. Ultimately, vascular access should be managed not only as a route for dialysis delivery but as a modifiable determinant of long-term cardiovascular health, one that benefits from early recognition of excessive flow, thoughtful planning, and timely flow-modulating interventions.
